# The Role of Endothelial Ca^2+^ Signaling in Neurovascular Coupling: A View from the Lumen

**DOI:** 10.3390/ijms19040938

**Published:** 2018-03-21

**Authors:** Germano Guerra, Angela Lucariello, Angelica Perna, Laura Botta, Antonio De Luca, Francesco Moccia

**Affiliations:** 1 Department of Medicine and Health Sciences “Vincenzo Tiberio”, University of Molise, via F. De Santis, 86100 Campobasso, Italy; germano.guerra@unimol.it (G.G.); angelicaperna@gmail.com (A.P.); 2 Department of Mental Health and Preventive Medicine, Section of Human Anatomy, University of Campania “L. Vanvitelli”, 81100 Naples, Italy; angela.lucariello@gmail.com (A.L.); antonio.deluca@unicampania.it (A.D.L.); 3 Laboratory of General Physiology, Department of Biology and Biotechnology “L. Spallanzani”, University of Pavia, via Forlanini 6, 27100 Pavia, Italy; laura.botta@unipv.it

**Keywords:** neurovascular coupling, neuronal activity, brain endothelial cells, Ca^2+^ signaling, glutamate, acetylcholine, ATP, nitric oxide, endothelial-dependent hyperpolarization, TRP channels

## Abstract

Background: Neurovascular coupling (NVC) is the mechanism whereby an increase in neuronal activity (NA) leads to local elevation in cerebral blood flow (CBF) to match the metabolic requirements of firing neurons. Following synaptic activity, an increase in neuronal and/or astrocyte Ca^2+^ concentration leads to the synthesis of multiple vasoactive messengers. Curiously, the role of endothelial Ca^2+^ signaling in NVC has been rather neglected, although endothelial cells are known to control the vascular tone in a Ca^2+^-dependent manner throughout peripheral vasculature. Methods: We analyzed the literature in search of the most recent updates on the potential role of endothelial Ca^2+^ signaling in NVC. Results: We found that several neurotransmitters (i.e., glutamate and acetylcholine) and neuromodulators (e.g., ATP) can induce dilation of cerebral vessels by inducing an increase in endothelial Ca^2+^ concentration. This, in turn, results in nitric oxide or prostaglandin E2 release or activate intermediate and small-conductance Ca^2+^-activated K^+^ channels, which are responsible for endothelial-dependent hyperpolarization (EDH). In addition, brain endothelial cells express multiple transient receptor potential (TRP) channels (i.e., TRPC3, TRPV3, TRPV4, TRPA1), which induce vasodilation by activating EDH. Conclusions: It is possible to conclude that endothelial Ca^2+^ signaling is an emerging pathway in the control of NVC.

## 1. Introduction

The brain comprises only 2% of the total body mass, yet it accounts for 20% of the overall energy metabolism [1]. As the brain has a limited capacity to store energy and lacks survival mechanisms that render other organs, such as heart and liver, more tolerant to short periods of anoxia or ischemia, the continuous supply of oxygen (O_2_) and nutrients and removal of catabolic waste are critical to maintain neuronal integrity and overall brain function. Accordingly, the brain receives up to 20% of cardiac output and consumes ≈20% and ≈25% of total body’s O_2_ and glucose [2,3,4]. Brain functions cease within seconds after the interruption of cerebral blood flow (CBF), while irreversible neuronal injury occurs within minutes [2,3]. Cerebral autoregulation is the mechanism whereby CBF remains relatively stable in spite of physiological fluctuations in arterial pressure, at least within a certain range. Thus, cerebral arteries constrict in response to an increase in arterial pressure and relax upon a decrease in blood pressure [4,5]. A subtler mechanism, known as functional hyperemia or neurovascular coupling (NVC), intervenes to locally increase the rate of CBF to active brain areas, thereby ensuring adequate matching between the enhanced metabolic needs of neural cells and blood supply [2,4,6]. Through NVC, a local elevation in neuronal activity (NA) causes a significant vasodilation of neighboring microvessels, which increases CBF and generates the blood oxygenation level-dependent (BOLD) signals that are used to monitor brain function through functional magnetic resonance imaging [7,8]. NVC is finely orchestrated by an intercellular signaling network comprised of neurons, astrocytes and vascular cells (endothelial cells, smooth muscle cells and pericytes), which altogether form the neurovascular unit (NVU) [4,6,9]. Synaptically-activated neurons may signal to adjacent vessels either directly or through the interposition of glial cells. Recent evidence has shown that astrocytes modulate NVC at arteriole levels, whereas they mediate neuronal-to-vascular communication at capillaries [2,4,10,11,12,13,14]. NA controls cerebrovascular tone through a number of Ca^2+^-dependent vasoactive mediators, which regulate the contractile state of either vascular smooth muscle cells (VSMCs) (arteries and arterioles) or pericytes (capillaries). These include nitric oxide (NO) and the arachidonic acid (AA) derivatives, prostaglandin E2 (PGE2), epoxyeicosatrienoic acids (EETs) and (20-HETE) [2,4,9]. While the role played by astrocytes and mural cells, i.e., VSMCs and pericytes, in CBF regulation has been extensively investigated, the contribution of microvascular endothelial cells to NVC has been largely underestimated [7]. This is quite surprising as the endothelium regulates the vascular tone in both systemic and pulmonary circulation by lining the innermost layer of all blood vessels [15,16,17]. Endothelial cells decode a multitude of chemical (e.g., transmitters and autacoids) and physical (e.g., shear stress pulsatile stretch) signals by generating spatio-temporally-patterned intracellular Ca^2+^ signals, which control VSMC contractility by inducing the synthesis and release of the vasorelaxing factors, NO, prostacyclin (or PGI2), carbon monoxide and hydrogen sulfide, as well as the vasoconstricting prostaglandin H2 and thromboxane A2 [15,18,19,20,21]. Moreover, an increase in sub-membranal Ca^2+^ concentration stimulates intermediate- and small-conductance Ca^2+^-activated K^+^ channels (IK_Ca_ and SK_Ca_, respectively), thereby causing an abrupt hyperpolarization in endothelial membrane potential, which is electronically transmitted to adjacent VSMCs and causes vessel dilation [16,22,23]. Herein, we aim at surveying the role of endothelial Ca^2+^ signaling in NVC by examining the physiological stimuli, e.g., neurotransmitters, neuromodulators and dietary molecules, that modulate CBF through an increase in intracellular Ca^2+^ concentration ([Ca^2+^]_i_) in brain microvascular endothelial cells.

## 2. Neurovascular Coupling

### 2.1. Cerebral Circulation and the Neurovascular Unit

**Figure 1:**
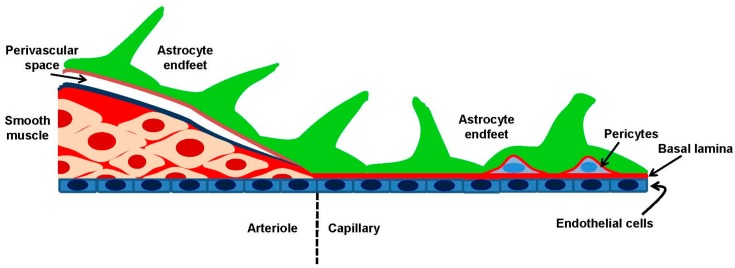
Cellular composition of the neurovascular unit. The vascular wall presents a different structure in arterioles and capillaries, which control the local supply of cerebral blood. Smooth muscle cells form one or more continuous layers around arterioles and change in their contractile state determine vessel diameter and regulate blood perfusion. Capillary diameter is regulated by contractile pericytes, which extend longitudinally and circumferentially along the capillary wall. Astrocyte end feet envelope arterioles and capillaries and are able to release vasoactive mediators, which regulate the contractile state of smooth muscle cells (arterioles) and pericytes (capillary) in response to neuronal activity.

The concept of NVU was first introduced during the first Stroke Progress Review Group meeting of the National Institute of Neurological Disorders and Stroke of the NIH (July 2001) to highlight the relevance to CBF regulation of the intimate association between neurons and cerebral vessels [4]. The NVU represents a functional unit where neurons, interneurons and astrocytes are in close proximity and are functionally coupled to smooth muscle cells, pericytes, endothelial cells and extracellular matrix (Figure 1). Within the NVU, neurons, glial and vascular cells establish a mutual influence on each other to ensure a highly efficient system to match blood and nutrient supply to the local needs of brain cells [2,4,6]. The interaction between neurons, glial cells and blood vessels may, however, vary along the cerebrovascular tree. Large cerebral arteries arise from Circle of Willis at the base of the brain and give rise to a heavily-interconnected network of pial arteries and arterioles, which run along the cortical surface. Cerebral vessels are lined by a single layer of endothelial cells (*tunica intima*), which is separated from an intermediate layer of smooth muscle cells (*tunica media*) by the internal elastic lamina. An outermost layer mainly comprised of collagen fibers and fibroblasts and enriched in perivascular nerves, known *as tunica adventitia*, is separated from the brain by the Virchow–Robin space, which is an extension of the sub-arachnoid space [4,6]. Penetrating arterioles branch off smaller pial arteries and dive into the brain tissue, thereby giving rise to parenchymal arterioles. The muscular component of the vascular wall is reduced to a single layer of VSMCs in intracerebral arterioles: of note, endothelial cells may project through the internal elastic lamina by establishing direct connections, known as myoendothelial projections, with the adjoining smooth muscle cells. Myoendothelial projections are enriched with gap junctions and allow the bidirectional transfer of information between endothelial cells and VSMCs [24]. While penetrating arterioles are still separated from the substance of the brain by the Virchow–Robin space, perivascular astrocytic processes (i.e., end feet) enter in contact and fuse together with the basal lamina of parenchymal arterioles, so that the perivascular space is obliterated [4,6]. Relevant to local CBF regulation, pyramidal cells and γ-aminobutyric acid (GABA) interneurons provide extensive innervation to parenchymal arterioles, often with the interposition of glial cells [4,10,25,26,27,28]. In addition, sub-cortical neurons from locus coeruleus, raphe nucleus and basal forebrain may also send projection fibers, containing respectively acetylcholine, norepinephrine and 5-hydroxytryptamine (5-HT), to intracortical microvessels and surrounding astrocytes [29]. Finally, parenchymal arterioles supply the cerebral circulation by giving rise to a dense network of intercommunicating capillary vessels, which are composed only of specialized endothelial cells and lack VSMCs. However, ≈30% of the brain capillary surface is covered by spatially-isolated contractile cells, i.e., pericytes, while the remaining endothelial capillary tubes are almost entirely wrapped by astrocytic end feet [30,31]. Astrocytes and pericytes located outside of brain capillaries may be innervated by local neurons, as described for astrocytes and VSMCs situated in the upper districts of the vascular tree [2]. The average capillary density of human brain amounts to ≈400 capillaries mm^−2^, which is enough to ensure that each neuron is endowed with its own capillary and to reduce on average to less than 15 μM the diffusion distance for O_2_, nutrients and catabolic waste [32,33]. Therefore, brain microvascular endothelial cells are ideally positioned to sense neurotransmitters released by axonal terminals during local synaptic activity and by sub-cortical projections provided that they express their specific membrane receptors. The following increase in [Ca^2+^]_i_ could, in turn, drive the synthesis of Ca^2+^-dependent vasoactive mediators. At the same time, brain microvascular endothelial cells have the potential to regulate local CBF independently of NA by detecting changes in blood flow variations and in the concentration of blood-borne agonists through distinct patterns of intracellular Ca^2+^ elevation.

### 2.2. Cellular and Biochemical Pathways of Neurovascular Coupling: The Role of Neurons and Astrocytes in Arterioles and Capillaries

**Figure 2:**
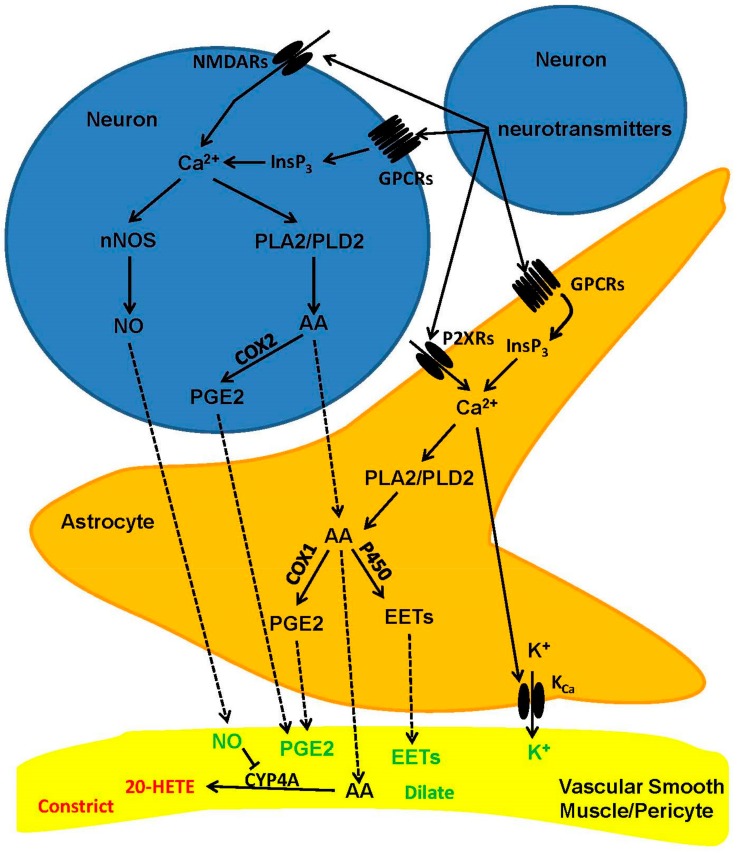
The mechanisms by which neurons and astrocytes stimulate arteriole and capillary dilation in response to synaptic activity. Synaptic activity increases intracellular Ca^2+^ levels within the postsynaptic neuron by stimulating metabotropic G-protein-coupled receptors (GPCRs), ionotropic receptors (e.g., NMDARs) or L-type voltage-operated Ca^2+^ channels (VOCs). This increase in [Ca^2+^]_i_ leads to the synthesis of NO and PGE2, which may relax both smooth muscle cells (arterioles) and pericytes (capillaries). Synaptically-released neurotransmitters may also increase [Ca^2+^]_i_ in perisynaptic astrocytes, thereby triggering NO release and PGE2/EET production. AA, which may be synthesized by PLA2 in both neurons and astrocytes, may be converted in the vasoconstricting factor, 20-HETE, in perivascular cells. Abbreviations: 20-HETE: 20-hydroxyeicosatetraenoic acid; AA: arachidonic acid; COX1: cyclooxygenase 1; COX2: cyclooxygenase 2; EETs: epoxyeicosatrienoic acids; GPCRs; G-protein-coupled receptors; InsP_3_: inositol-1,4,5-trisphosphate; K_Ca_: Ca^2+^-activated intermediate and small conductance K^+^ channels; NO: nitric oxide; nNOS: neuronal NO synthase; P450: cytochrome P450; PGE2: prostaglandin E2; PLA2: phospholipase A2; PLD2: phospholipase D2; VOCs: L-type voltage-operated Ca^2+^ channels.

The mechanisms whereby synaptic activity controls the microvascular tone vary depending on the brain structure and may differ along the vascular tree (Figure 2) [2,4,9]. An increase in intracellular Ca^2+^ concentration ([Ca^2+^]_i_) within the dendritic tree is the crucial signal that triggers the synthesis and release of vasoactive messengers in response to excitatory synaptic inputs [2,4,9,34]. For instance, glutamate stimulates post-synaptic *N*-methyl-d-aspartate (NMDA) and a-amino-3-hydroxy-5-methyl-4-isoxazol epropionic acid (AMPA) receptors to induce extracellular Ca^2+^ entry and recruit the Ca^2+^/calmodulin (Ca^2+^/CaM)-dependent neuronal nitric oxide (NO) synthase (nNOS) in hippocampal and cerebellar GABA interneurons [10,25,28,33,35]. The following NO release elicits arteriole vasodilation by inducing VSMC hyperpolarization and relaxation through a soluble guanylate cyclase/protein kinase G (PKG)-dependent mechanism (see below) [13,26,35,36,37]. Conversely, NMDA receptor (NMDAR)-mediated Ca^2+^ entry in synaptically-activated pyramidal neurons of the somatosensory cortex engages cyclooxygenase 2, which catalyzes the synthesis of the powerful vasodilator, prostaglandin E2 (PGE2), which acts through EP2 and EP4 receptors on VSMCs [27,28]. Intriguingly, NO plays a permissive role in PGE2-dependent vasodilation by maintaining the hemodynamic response during sustained NA [25,38]. Long-lasting synaptic activity could lead to an increase in [Ca^2+^]_i_ within perisynaptic astrocytic processes (Figure 2), which lags behind the onset of CBF, but is able to activate phospholipase A2 (PLA2) and cleave AA from the plasma membrane. AA, in turn, diffuses to adjacent VSMCs and is converted into the vasoconstricting messenger, 20-hydroxyeicosatetraenoic (20-HETE), by cytochrome P450 4A (CYP4A) [33,38]. However, neuronal-derived NO inhibits CYP4A, thereby preventing 20-HETE formation and maintaining PGE2-dependent vasodilation [9,33,38]. The mechanism(s) whereby NA induces astrocytic Ca^2+^ signals is still unclear. Glutamate has been predicted to increase astrocyte [Ca^2+^]_i_ by binding to metabotropic glutamate receptors (mGluRs) 1 and 5 (mGluR1 and mGluR5, respectively), which stimulate phospholipase Cβ (PLCβ) through Gqα monomer and induce inositol-1,4,5-trisphosphate (InsP_3_)-dependent Ca^2+^ release from the endoplasmic reticulum (ER) [9,39]. However, mGluR1 and mGluR5 are lacking in adult astrocytes, and the genetic deletion of type 2 InsP_3_ receptor (InsP_3_R2), which represents the primary InsP_3_R isoform in glial cells, does not prevent NVC [2,30,39]. Nevertheless, there is indisputable evidence that astrocytes require mGluRs to drive the hemodynamic response to sensory stimulation also in adult mice [24,40,41]. Furthermore, alternative mechanisms may drive astrocyte Ca^2+^ signaling, including NMDARs, purinergic receptors and multiple transient receptor potential (TRP) channels [14,39]. We refer the reader to a number of exhaustive and recent reviews about the controversial role of astrocyte [Ca^2+^]_i_ in NVC [14,30,39,42]. Intriguingly, astrocytes are able to sense transmural pressure across the vascular wall through vanilloid TRP 4 (TRPV4) channels [43,44], which are located on their perivascular end feet. It has, therefore, been proposed that the initial hemodynamic response to NA activates these mechanosensitive Ca^2+^-permeable channels, thereby causing an increase in astrocyte [Ca^2+^]_i_ and recruiting the Ca^2+^-dependent PLA2 [14]. In addition, synaptically-released ATP could mobilize ER Ca^2+^ by activating P2Y2 and P2Y4 receptors, which are located on astrocytic end feet wrapped around cerebral vessels [45].

Although it has long been thought that CBF regulation occurs at the arteriole level [6], recent studies have convincingly shown that most of the hydraulic resistance that must be decreased in order to increase cortical perfusion is located in the capillary bed, which are wrapped by contractile pericytes [30,46,47]. This model makes physiological sense as, on average, firing neurons are remarkably closer to capillaries than to arterioles (8–23 μm away versus 70–160 μm) [47]. Therefore, capillaries are located in a much more suitable position to rapidly detect NA and initiate the hemodynamic response that ultimately generates BOLD signals. In addition, the regulation of CBF at the capillary level could selectively increase CBF only in active areas, thereby finely matching the local tissue O_2_ supply to local cerebral demand [30,46,47]. The cerebellar cortex is composed of three layers: molecular layer, Purkinje cells and granular layer, respectively, from outermost to innermost [48]. Recent studies demonstrated that, at the molecular layer, synaptic activity caused an increase in astrocytic end feet Ca^2+^ concentration by inducing Ca^2+^ entry through P2X1 channels. This spatially-restricted Ca^2+^ signal, in turn, recruited phospholipase D2 (PLD2) and diacylglycerol lipase to synthesize AA, which was then metabolized by cyclooxygenase 1 into PGE2. Finally, PGE2 evoked capillary dilation by binding to EP4 receptors, which were presumably located on pericytes [13]. Conversely, in the granular layer, synaptic activity induced robust NO release by promoting NMDARs-mediated Ca^2+^ entry in granule cells without astrocyte involvement [11]. 

### 2.3. Is There a Role for Endothelial Ca^2+^ Signaling in Neurovascular Coupling?

Vascular endothelial cells control vascular tone by releasing a myriad of vasoactive mediators in response to an increase in [Ca^2+^]_i_ evoked by either chemical or mechanical inputs [16,18,19,20,49]. For instance, endothelial Ca^2+^ signals trigger the selective increase in blood flow to skeletal, respiratory and cardiac muscles induced by physical exercise/training [15]. Curiously, it is still unclear whether and how endothelial Ca^2+^ signals play any role in translating NA into vasoactive signals in the brain [7,24]. Luminal perfusion of neurotransmitters and neuromodulators, such as acetylcholine, ATP, ADP and bradykinin, results in the dilation of cerebral arterioles by inducing the activation of specific Gq-protein-coupled receptors (GPCRs), which are located on the endothelial membrane [29,50,51,52,53]. GPCRs stimulate PLCβ to synthesize the ER Ca^2+^-releasing messenger, InsP_3_, thereby triggering the Ca^2+^-mediated signaling cascade that leads to the synthesis and release of most endothelial-derived vasoactive messengers [16,18,19,20,49,54]. Nevertheless, only scarce information is available about the role of endothelial Ca^2+^ signaling in the hemodynamic response to NA. As described elsewhere [50], synaptically-activated neurons and/or astrocytes could directly stimulate brain microvessels by releasing mediators that traverse the VSMC or pericyte layers and bind to receptors located on the abluminal side of endothelium. Interestingly, brain microvascular endothelial cells may also express NMDARs [55,56] and mGluR1 [57,58,59,60], although their potential contribution to NVC has been barely appreciated [4]. Accordingly, recent studies demonstrated that endothelial NMDARs participate in glutamate-induced vasodilation of intraparenchymal arterioles by mediating Ca^2+^ entry and subsequent eNOS activation [55,56]. Finally, brain endothelium could actively mediate the retrograde propagation of the vasodilation signal from the capillaries feeding the sites of NA to the upstream pial vessels (arteries and arterioles) that supply the activated area [6,7,61]. Retrograde vasodilation into the proximal arterial supply is required to achieve an optimally-localized increase in blood flow to active neurons and to avoid a ‘‘flow steal’’ from interconnected vascular networks [4,62]. Retrograde propagation in peripheral vessels is accomplished by endothelial Ca^2+^ signals that impinge on two distinct components to drive the conducted vasomotor response: (1) the Ca^2+^-dependent rapid activation of EDH, which is restricted to stimulated endothelial cells, but is rapidly transmitted to more remote sites along the vessel and (2) a slower intercellular Ca^2+^ wave that spreads vasodilation via endothelial release of NO and PGE2 [7,63,64]. In the following section, we will discuss the evidence supporting the contribution of endothelial Ca^2+^ signals in NVC and the possibility that interendothelial Ca^2+^ waves are involved in the control of cerebrovessel diameters and microhemodynamics during intense synaptic activity.

## 3. The Role of Endothelial Ca^2+^ Signaling in Neurovascular Coupling

### 3.1. Endothelial Ca^2+^ Signaling in Brief

**Figure 3:**
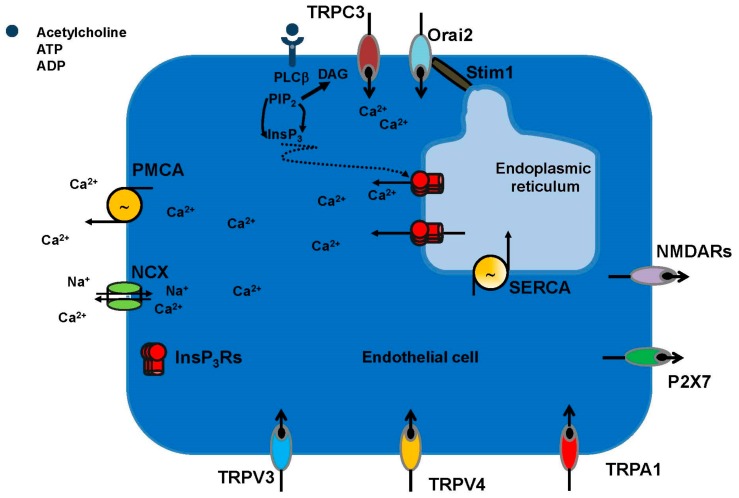
The Ca^2+^ signaling toolkit in brain microvascular endothelial cells. There is scarce information available regarding the molecular components of the Ca^2+^ signaling toolkit in brain microvascular endothelial cells. A recent investigation, however, provided a thorough characterization of the Ca^2+^ machinery in bEND5 cells [76], which represent an established mouse brain microvascular endothelial cell line. Further information was obtained by the analysis of endothelial Ca^2+^ signals in rodent parenchymal arterioles and in the human hCMEC/D3 cell line. Extracellular autacoids bind to specific G-protein-coupled receptors, such as M-AchRs and P2Y1 receptors, thereby activating PLCβ, which in turn cleaves PIP_2_ into InsP_3_ and DAG. InsP_3_ triggers ER-dependent Ca^2+^ releasing by gating InsP_3_Rs, while DAG could activate TRPC3. The InsP_3_-dependent drop in ER Ca^2+^ levels induces SOCE, which is mediated by the interaction between Stim1 and Orai2 in bEND5 cells. Moreover, extracellular Ca^2+^ entry may occur through TRPV3, TRPV4 and TRPA1, which are coupled to either eNOS or EDH [77,78]. Finally, brain microvascular endothelial cells may express Ca^2+^-permeable ionotropic receptors, such as NMDARs and P2X7 receptors. The elevation in [Ca^2+^]_i_ decays to the baseline via the concerted interaction between SERCA and PMCA pumps, as well as through NCX [72,79,80]. Abbreviations: InsP_3_, inositol-1,4,5-trisphosphate; DAG, diacylglycerol; InsP_3_Rs, InsP_3_ receptors; NCX, Na^+^–Ca^2+^ exchanger; PMCA, plasma membrane Ca^2+^ ATPase; PIP_2_, phosphatidylinositol-4,5-bisphosphate; PLCβ, phospholipase Cβ; SERCA, sarco-endoplasmic reticulum Ca^2+^-ATPase. The thicker line connecting PIP_2_ to InsP_3_ indicates a high amount of second messenger produced upon PLCβ activation.

It has long been known that an increase in endothelial [Ca^2+^]_i_ delivers the crucial signal to induce the synthesis of multiple vasoactive mediators [16,17,18,19,20]. For instance, intracellular Ca^2+^ signals recruit the Ca^2+^/CaM-dependent endothelial NOS (eNOS) and the Ca^2+^-dependent phospholipase A2 (PLA_2_) to generate NO and prostaglandin I2 (PGI_2_ or prostacyclin), respectively [16,19]. The Ca^2+^ response to extracellular autacoids typically consists of an initial Ca^2+^ peak, which is due to InsP_3_-dependent ER Ca^2+^ release, followed by sustained Ca^2+^ entry through store-operated Ca^2+^ channels (Figure 3) [65,66,67]. Store-operated Ca^2+^ entry (SOCE) is a major Ca^2+^ entry pathway in endothelial cells, being activated by any stimulus leading to the depletion of the ER Ca^2+^ pool [68,69,70]. The dynamic interplay between InsP_3_-dependent Ca^2+^ release, which could be amplified by adjoining ryanodine receptors (RyRs) through the process of Ca^2+^-induced Ca^2+^ release (CICR) (Figure 3), and SOCE results in biphasic Ca^2+^ signals [71,72] or repetitive [Ca^2+^]_i_ oscillations in brain vascular endothelium [73,74,75]. 

#### 3.1.1. The Endothelial Ca^2+^ Toolkit in Brain Microvascular Endothelial Cells: Endogenous Ca^2+^ Release

Scarce information is available regarding the composition of the Ca^2+^ toolkit in brain endothelial cells [74]. For instance, human brain microvascular endothelial cells expressed InsP_3_R1 and displayed ER-dependent Ca^2+^ release in response to an increase in InsP_3_ levels [81]. Conversely, only InsP_3_R2 was expressed in capillary endothelium of rat hippocampus, while RyRs could not be detected in the same study [82]. Nevertheless, RyRs sustained InsP_3_-dependent Ca^2+^ release in rat brain microvascular endothelial cells in vitro [71], which suggests that the brain endothelial Ca^2+^ toolkit could undergo substantial remodeling in cell cultures [19]. A recent investigation carried out a thorough investigation of the Ca^2+^ toolkit in bEND5 cells [76], a widely-employed mouse brain microvascular endothelial cell line [83]. This analysis revealed that bEND5 cells expressed both InsP_3_R1 and InsP_3_R2, while they lacked InsP_3_R2 and RyRs [76]. ER-mobilized Ca^2+^ may lead to an increase in mitochondrial Ca^2+^ concentration due to the physical interaction between InsP_3_Rs and the Ca^2+^-permeable voltage-dependent anion channels (VDACs), which are embedded in the outer mitochondrial membrane. InsP_3_Rs-released Ca^2+^ is transferred by VDAC1 into the intermembrane space, from which it is routed towards the mitochondrial matrix though the mitochondrial Ca^2+^ uniporter (MCU) [84,85]. This ER-to-mitochondria Ca^2+^ transfer boosts cellular bioenergetics by stimulating intramitochondrial Ca^2+^-dependent dehydrogenases, such as oxoglutarate dehydrogenase, NAD-isocitrate dehydrogenase and pyruvate dehydrogenase [86,87,88]. Subsequently, endothelial mitochondria may contribute to silently (i.e., without a global increase in [Ca^2+^]_i_) refill the ER in a sarco-endoplasmic reticulum Ca^2+^-ATPase (SERCA)-mediated fashion by releasing Ca^2+^ though the mitochondrial Na^+^/Ca^2+^ exchanger [89,90]. Ca^2+^ entry through the MCU is driven by the negative (i.e., −180 mV) membrane potential (ΔΨ) that exists across the inner mitochondrial membrane [86]. Mitochondrial content in cerebrovascular endothelium is significantly higher as compared to other vascular districts [91], and InsP_3_Rs-mediated mitochondrial Ca^2+^ signals arise in rat [92,93] and human [94] brain capillary endothelial cells. Moreover, mitochondrial depolarization hampers the ER-to-mitochondrial Ca^2+^ shuttle, thereby causing a remarkable increase in [Ca^2+^]_i_ in rat brain microvascular endothelial cells [95]. An additional mode of Ca^2+^-mediated cross-talk in endothelial cells may be established between ER and the acidic vesicles of the endolysosomal (EL) Ca^2+^ store [96,97]. The Ca^2+^-releasing messenger, nicotinic acid adenine dinucleotide phosphate (NAADP), mobilizes EL Ca^2+^ by gating two-pore channels 1 (TPC1) or TPC2 in several types of endothelial cells [54,98,99,100]. Lysosomal Ca^2^ release may be amplified by adjacent ER-embedded InsP_3_Rs through CICR [97]. The role of lysosomal Ca^2+^ signaling in brain microvascular endothelial cells remains, however, elusive.

#### 3.1.2. The Endothelial Ca^2+^ Toolkit in Brain Microvascular Endothelial Cells: Endogenous Ca^2+^ Release

Likewise, the molecular structure of SOCE in brain vascular endothelium remains to be fully elucidated. Typically, endothelial SOCE is comprised of Stromal interaction molecule 1 and 2 (Stim1 and Stim2, respectively), which sense the drop in ER Ca^2+^ concentration ([Ca^2+^]_ER_) and Orai1-2 channels, which provide the Ca^2+^-permeable channel-forming subunits on the plasma membrane [65,76,101,102,103,104]. More specifically, Stim2 is activated by small fluctuations in [Ca^2+^]_ER_, drives resting Ca^2+^ entry and maintains basal Ca^2+^ levels in endothelial cells [103], while Stim1 is engaged by massive ER Ca^2+^ depletion and sustains agonists-induced extracellular Ca^2+^ entry [65,76,101,102,104]. However, exceptions to this widespread model may exist. For instance, Stim2 expression is rather modest, and Orai2 represents the only Orai isoform endowed to mouse brain microvascular cells [76]. Of note, Orai2 constitutes the prominent pore-forming subunit of store-operated channels also in mouse neurons [105], which is consistent with the notion that endothelial cells are sensitive to both environmental cues and epigenetic modifications [106]. As widely illustrated in [106,107], the endothelial phenotype is unique in its plasticity and depends on both site-specific signal inputs delivered by the surrounding milieu (which may be diluted in cell culture) and by site-specific epigenetic modifications (i.e., DNA methylation, histone methylation and histone acetylation), which persist under in vitro culture conditions. Similar to other endothelial cells types [108,109], SOCE is constitutively activated to refill the InsP_3_-dependent ER Ca^2+^ pool and maintains basal Ca^2+^ levels in mouse brain microvascular endothelial cells [76]. AA may induce extracellular Ca^2+^ entry in vascular endothelial cells by prompting Orai1 to interact with Orai3 independently on Stim1 [110]. Nevertheless, Orai3 is not expressed in bEND5 cells, and therefore, this mechanism is unlikely to work in NVC [76]. The endothelial SOCE machinery could involve additional components, such as members of the canonical TRP (TRPC) sub-family of non-selective cation channels, of which seven isoforms exist (TRPC1-7) [68,111]. For instance, Stim1 could also recruit TRPC1 and TRPC4 to assemble into a ternary complex [67,112], whose Na^+^/Ca^2+^ permeability is determined by Orai1 [113,114]. TRPC1 was expressed in bEND5 cells, while TRPC4 was absent [76]. However, TRPC1 requires Orai1 to be recruited into the SOCE complex upon ER Ca^2+^ depletion [112,115]. It is, therefore, unlikely that it contributes to SOCE in mouse brain microvascular endothelial cells, which lack Orai1 [76], while it may assemble with polycystic TRP2 (TRPP2) to form a stretch-sensitive Ca^2+^-permeable channel [116]. In addition to SOCE, PLCβ-dependent signaling may lead to the activation of diacylglycerol (DAG)-sensitive Ca^2+^ channels, such as TRPC3 [117,118] and TRPC6 [119,120]. The TRP superfamily of non-selective cation channels consists of 28 members that are classified into six sub-families based on their amino acid sequence homology and structural homology [121,122]. These subfamilies are designated as canonical (TRPC1-7), vanilloid (TRPV1-6), melastatin (TRPM1-8), ankyrin (TRPA1), mucolipin (TRPML1-3) and polycystin (TRPP; TRPP2, TRPP3 and TRPP5) [78,121]. Endothelial cells from different vascular beds may dispose of distinct complements of TRP channels to respond to a myriad of different chemical, mechanical and thermal stimuli [19,78,123,124]. Vascular tone in the brain is specifically regulated by a restricted number of TRP channels, including TRPC3, TRPV3, TRPV4 and TRPA1 (Figure 3), which may stimulate NO release and/or activate IK_Ca_ and SK_Ca_ channels to trigger endothelial-dependent hyperpolarization (EDH) [19,77,78,124]. Endothelial TRPV4 has a potentially relevant role in NVC as it can be activated by AA and its cytochrome P450 epoxygenases-metabolites, i.e., epoxyeicosatrienoic acids (EETs), which evoke vasodilation in intraparenchymal arterioles [9,125]. Finally, human [126], mouse [55,56,127] and rat [128] brain endothelial cells express functional NMDARs (Figure 3) [55], which may mediate glutamate-induced extracellular Ca^2+^ entry. Likewise, P2X7 receptors were recently found in both hCMEC/D3 cells (Figure 3) [129], an immortalized human brain endothelial cell line, and in rat brain endothelial cells in situ [130]. Therefore, the multifaceted endothelial Ca^2+^ toolkit, being located at the interface between neuronal projections and flowing blood, is in an ideal position to trigger and/or modulate NVC by sensing synaptically-released neurotransmitters and blood-borne autacoids.

### 3.2. Endothelial NMDA Receptors Trigger Glutamate-Induced Nitric Oxide-Mediated Vasodilation

**Figure 4:**
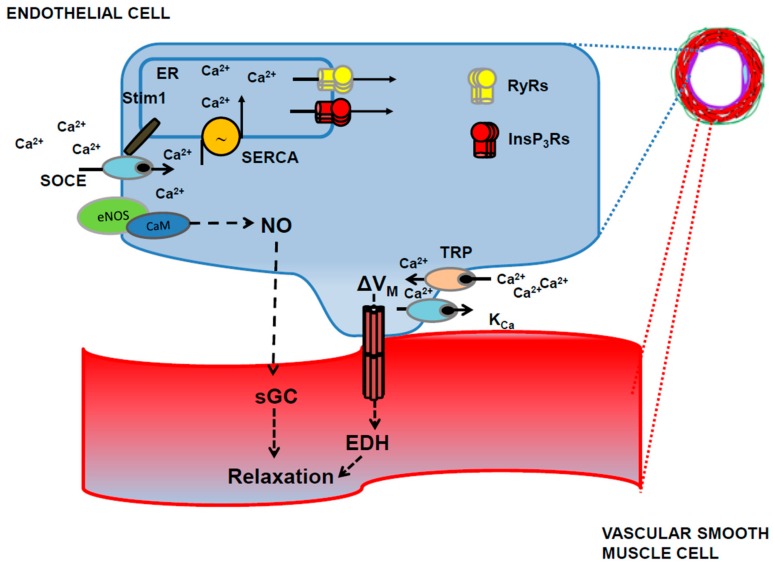
Ca^2+^-regulated endothelium-dependent vasodilation. Agonists-induced InsP_3_-dependent ER Ca^2+^ depletion in vascular endothelial cells leads to store-operated Ca^2+^ entry (SOCE). SOCE is tightly coupled to eNOS, thereby triggering robust NO release. NO, in turn, diffuses towards adjacent vascular smooth muscle cells (VSMCs) at myo-endothelial projections and activates soluble guanylyl cyclase (sGC) to induce vasorelaxation. Extracellular agonists may also activate TRP channels (e.g., TRPC3, TRPV3, TRPV4 and TRPA1), which is preferentially coupled to Ca^2+^-activated K^+^ channels (K_Ca_), such as IK_Ca_ and SK_Ca_. Endothelial hyperpolarization spreads through myo-endothelial gap junctions to adjoining VSMCs to induce vasorelaxation according to a mechanism known endothelial-dependent hyperpolarization (EDH).

NO represents the major mediator whereby endothelial cells control the vascular tone in large conduit arteries (up to 100%), while its contribution to agonists and/or flow-induced vasodilation decreases (up to 20–50%) as the vascular tree branches into the network of arterioles and capillaries that locally control blood flow [131,132]. NO release is mainly sustained by SOCE rather than by ER-dependent Ca^2+^ release (Figure 4) [133,134,135], and an oscillatory increase in [Ca^2+^]_i_ is the typical waveform that leads to extracellular autacoids-induced NO liberation from vascular endothelial cells [136,137]. In addition to SOCE, TRPC3 [138] and TRPV4 [139] may also evoke extracellular Ca^2+^ entry-mediated eNOS activation, NO release and endothelium-dependent vasodilation. Once released, NO diffuses to adjoining VSMCs to stimulate soluble guanylate cyclase and induce cyclic guanosine-3′,5′-monophosphate (cGMP) production (Figure 4). Then, cGMP activates protein kinase (PKG), which phosphorylates multiple targets to prevent the Ca^2+^-dependent recruitment of myosin light chain kinase and induce VSMC relaxation. For instance, PKG-dependent phosphorylation inhibits the increase in VSMC [Ca^2+^]_i_ induced by L-type voltage-operated Ca^2+^ channels and InsP_3_Rs; in addition, PKG phosphorylates SERCA, thereby boosting cytosolic Ca^2+^ sequestration into the ER lumen [16,140]. In addition, PKG-dependent phosphorylation stimulates large-conductance Ca^2+^-activated K^+^ channels (BK_Ca_), thereby inducing VSMC hyperpolarization and vessel dilation [16,140]. Finally, NO accelerates SERCA-mediated Ca^2+^ reuptake through S-nitrosylation of its cysteine thiols [140]. It is generally assumed that neuronal-derived NO induces vasodilation in the hippocampus and cerebellum [10,25,26,28,33,35,36,37] and plays a permissive role in PGE2-induced vasorelaxation [9,33,38]. However, alternative sources, e.g., eNOS, have been implicated in glutamate-induced NVC [141,142]. Moreover, glutamate evoked endothelium-dependent vasodilation in the presence of D-serine, a NMDAR co-agonist, in isolated middle cerebral arteries and in brain slice parenchymal arterioles [55,143,144]. NMDARs are heterotetramers comprising seven distinct subunits (GluN1, GluN2A–D and GluN3A and B); GluN1 is strictly required for the assembly of a functional channel and for correct trafficking of the other subunits [144,145,146]. NMDAR activation requires binding of glutamate to GluN1 and of a co-agonist, D-serine or glycine, to GluN2 [145]. Two GluN1 subunits associate with GluN2A and GluN2B to form neuronal NMDARs, which are therefore sensitive to extracellular Mg^2+^ block [145]. As a consequence, NMDAR activation requires simultaneous binding of synaptically-released glutamate and release of Mg^2+^ inhibition by AMPARs-dependent membrane depolarization [145]. However, in non-neuronal cells, GluN1 subunits assemble with GluN2C and GluN2D, which confer a lower sensitivity to Mg^2+^, while incorporation of GluN3 further decreases the inhibitory effect of extracellular Mg^2+^ and limits Ca^2+^ permeability [144,145]. NMDAR subunits (i.e., GluN1 and GluN2A-D) have been detected in brain endothelial cells in vitro [126,127,128,143,147,148] and in cerebral cortex in situ [127] (Figure 3). Intriguingly, NMDARs are more abundant on the basolateral endothelial membrane, which place them in the most suitable position to mediate direct neuronal-to-vascular communication [127]. Recently, Anderson’s group demonstrated that glutamate and NMDA induced vasodilation in isolated middle cerebral arteries and in brain slices penetrating arterioles only in the presence of the NMDAR co-agonist, D-serine [55]. This feature could explain why previous investigations, which omitted D-serine or glycine from the bathing solution, failed to observe NMDA-induced dilation of cerebral vessels [149]. Subsequently, the same group showed that astrocytic Ca^2+^ signals induced D-serine release, which was in turn able to activate endothelial NMDARs, thereby activating eNOS in a Ca^2+^-dependent manner [56,127]. Interestingly, NO evoked dilation of cortical penetrating arterioles by suppressing 20-hydroxyeicosatetraenoic acid (20-HETE) synthesis and boosting PGE2-induced vasorelaxation [56,127]. Future work will have to assess whether eNOS is also activated in response to somatosensory stimulation in vivo and to elucidate the physiological transmitter that induces the Ca^2+^ response in astrocytes. However, these findings clearly show that endothelial NMDARs may control CBF by engaging eNOS at the arteriole level.

### 3.3. Intracellular Ca^2+^ Signals Drive Acetylcholine-Induced Nitric Oxide Release from Brain Microvascular Endothelial Cells

The cortex receives a widespread acetylcholine innervation mainly arising from the basal forebrain nucleus [28,150]. Basal forebrain acetylcholine neurons broadly projects on intraparenchymal arterioles and capillaries and are, therefore, optimally suited to directly control CBF [28,29,150]. Accordingly, electrical stimulation of basal forebrain neurons results in dilation of intracortical arterioles and increases local CBF [53,151,152]. Acetylcholine-induced cerebral vasodilation is mediated by Gq-coupled muscarinic M5 receptors (M5-AchRs) [53,153], which activate eNOS and induce NO release from cerebrovascular endothelium, in mouse and pig [53,150]. Acetylcholine evokes NO release by triggering repetitive [Ca^2+^]_i_ oscillations in several types of endothelial cells [137,154,155]. A recent study focused on bEND5 cells to unravel how acetylcholine induces Ca^2+^-dependent NO release from cerebrovascular endothelium [76,156,157]. Acetylcholine triggered intracellular oscillations in [Ca^2+^]_i_, which were driven by rhythmical InsP_3_-dependent ER Ca^2+^ release and maintained by SOCE (Figure 3) [76]. The Ca^2+^ response to acetylcholine was mediated by M3-AchRs, which represent another PLCβ-coupled M-AchR isoform. Conversely, nicotine did not cause any detectable increase in [Ca^2+^]_i_ [76], as also observed in other endothelial cell types [158]. Acetylcholine-induced Ca^2+^ oscillations led to robust NO release, with eNOS requiring both intracellular Ca^2+^ release and extracellular Ca^2+^ entry to be fully activated [76]. These data were partially confirmed in hCMEC/D3 cells, which displayed a biphasic increase in [Ca^2+^]_i_ in response to acetylcholine [76]; of note, human brain microvascular endothelial cells express M5-AchRs [52]. Our preliminary data suggest that the distinct waveforms of acetylcholine-induced Ca^2+^ signals in human vs. mouse brain endothelial cells reflect crucial differences in their Ca^2+^ toolkit. Accordingly, hCMEC/D3 only express InsP_3_R3, while they lack InsP_3_R1 and InsP_3_R2 [159]). InsP_3_R2, which shows the sharpest dependence on ambient Ca^2+^ and is the most sensitive InsP_3_R isoform to InsP_3_, has long been known as the main oscillatory Ca^2+^ unit [160,161]. Conversely, InsP_3_R3, which is not inhibited by surrounding Ca^2+^, tends to suppress intracellular Ca^2+^ oscillations [160,161]. Therefore, endothelial Ca^2+^ signaling can be truly regarded as a crucial determinant for acetylcholine-induced NVC.

### 3.4. Endothelial Ca^2+^ Signals Could Mediate ATP-Induced Vasodilation

Following synaptic activity, neurons and astrocytes release ATP, which serves as a modulator of cellular excitability, synaptic strength and plasticity [162]. ATP is rapidly (200 ms) hydrolyzed by extracellular ectonucleotidases into ADP and adenosine [162], all these mediators being able to physiologically increase CBF [52,163]. Luminal application of ATP and ADP has long been known to induce endothelium-dependent vasodilation in isolated cerebral vessels. For instance, ATP and ADP promoted vasodilation in rat middle cerebral arteries by stimulating NO release, although ATP could also act through cytochrome P450-metabolites [51]. Of note, extraluminal administration of ATP evoked a biphasic vasomotor response in rat penetrating arterioles, consisting of an initial transient vasoconstriction followed by local vasodilation, which was subsequently propagated to upstream locations (≈500 μm) along the vascular wall [164,165]. ATP-induced local vasoconstriction was mediated by ionotropic P2X receptors on VSMCs, while endothelial P2Y1 receptors triggered local vasodilation [164]. ATP induced vasodilation by stimulating NO release and stimulating EET production to activate EDH (see below); EETs were also responsible for the upstream propagation of the vasomotor response [164]. These findings were supported by a recent study, showing that the hemodynamic response to whisker stimulation in the mouse somatosensory cortex required P2Y1 receptor-dependent eNOS activation. Previous administration of fluoroacetate, a glial-specific metabolic toxin, prevented eNOS-dependent functional hyperemia, thereby suggesting that ATP was mainly released by perivascular astrocytes [166]. P2Y1 receptors are GPCRs, which bind to both ATP and ADP and control the vascular tone by inducing intracellular Ca^2+^ signals in endothelial cells through distinct signaling pathways [167,168,169]. For instance, P2Y1 receptors induced InsP_3_-dependent ER Ca^2+^ release in rat cardiac microvascular endothelial cells [167], whereas they stimulated cyclic nucleotide-gated channels in the H5V endothelial cell line, in primary cultures of bovine aortic endothelial cells and in mouse aorta endothelial cells in situ [169]. Moreover, ATP induced local and conducted vasodilation in hamster cheek pouch arterioles by triggering an increase in endothelial [Ca^2+^]_i_ both locally and ≈1200 μm upstream along the same vessel (see also below) [170]. Therefore, it is conceivable that endothelial Ca^2+^ signals mediate P2Y1 receptor-dependent NO release and functional hyperemia in the somatosensory cortex. 

### 3.5. TRP Channels Trigger Endothelial-Dependent Hyperpolarization in Cerebrovascular Endothelial Cells

EDH provides the largest contribution to endothelial vasorelaxing mechanisms in resistance-sized arteries and arterioles, as shown in coronary, renal and mesenteric circulation [22,23,131,171]. EDH is initiated by an increase in endothelial [Ca^2+^]_i_, which stimulates IK_Ca_ (KCa_3.1_) and SK_Ca_ (KCa_2.3_) channels to hyperpolarize the endothelial cell membrane (Figure 4). Hyperpolarizing current spreads from vascular endothelium to overlying smooth muscle cells to trigger VSMC relaxation and vessel dilation by inhibiting voltage-dependent Ca^2+^ entry (Figure 4) [22,23,77]. This vasorelaxing mechanism requires a tightly-regulated disposition of the Ca^2+^ sources and the Ca^2+^-sensitive decoders, i.e., SK_Ca_ and IK_Ca_ channels, which effect membrane hyperpolarization. To achieve such a precise spatial arrangement, endothelial cells extend cellular protrusions through the internal elastic lamina, which establish a heterocellular coupling with adjacent smooth muscle cells through connexin-based myo-endothelial gap-junctions (MEGJs) [22,23,77]. The endothelial ER also protrudes into these myo-endothelial microdomain sites and forms spatially-discrete InsP_3_-sensitive Ca^2+^ pools, which are juxtaposed with IK_Ca_ channels, which are located on the endothelial protrusions traversing the holes in the vascular wall [23,172]. Conversely, SK_Ca_ channels are distributed throughout the endothelial cell membrane, although they are enriched at MEGJs and may, therefore, sense ER-released Ca^2+^ [173]. InsP_3_Rs are constitutively activated to produce repetitive, spatially-restricted Ca^2+^ release events, termed Ca^2+^ pulsars, whose frequency can be increased by extracellular vasoactive agonists, such as acetylcholine. Spontaneous InsP_3_-driven Ca^2+^ pulsars are selectively coupled to IK_Ca_ and SK_Ca_ channels, thereby hyperpolarizing the endothelial membrane potential at myo-endothelial projections and inducing dilation in adjoining VSMCs [172]. K^+^ signaling in the myo-endothelial space could then be boosted by the stimulation of endothelial inward rectifying K^+^ (K_ir_) channels or of Na^+^/K^+^ ATPase in VSMCs. In addition to InsP_3_-induced ER-dependent Ca^2+^ release, IK_Ca_ and SK_Ca_ channels can be activated by extracellular Ca^2+^ entry through TRPV4 channels, which is also largely expressed at MEGJs [174]. EDH does not only evoke local vasodilation at the site of endothelial stimulation; the hyperpolarizing current spreads along vascular endothelium to upstream arteries (up to ≈2 mm) and drives the retrograde vasodilation, which is ultimately responsible for the drop in vascular resistance that increases blood supply to active regions [175,176,177]. Preliminary data revealed that the same clustered architecture of TRPV4 and IK_Ca_ channels is maintained at MEGJs of rodent cerebral arteries [24,78,178,179,180], which suggests that discrete InsP_3_Rs-mediated Ca^2+^ pulsars arise also in parenchymal vessels [24]. Moreover, cerebral MEGJs are enriched with TRPA1 channels, which may also support EDH in cortical circulation [178,181]. EDH mediates ATP-, UTP- and SLIGR (a selective agonist of protease-activated receptor 2)-induced vasodilation in rat middle cerebral arteries [164,180,182,183,184,185] and acetylcholine-induced vasodilation in mouse posterior cerebral arteries [186].

#### 3.5.1. TRPV4

TRPV4 is emerging as a major regulator of CBF [24,39,163] and represents one of the most important Ca^2+^-entry pathways in vascular endothelial cells [111,187,188,189,190,191]. TRPV4 channels are polymodal non-selective cation channels that mediate Ca^2+^ entry in response to distinct chemical, thermal and mechanical stimuli [54,125,187,191,192,193,194], thereby integrating the diverse surrounding cues acting on the vascular wall. For instance, TRPV4 may be activated by EETs, which are produced via AA epoxygenation by cytochrome P450 epoxygenase enzymes. Briefly, an increase in endothelial [Ca^2+^]_i_ induced by either mechanical or chemical stimuli may stimulate the Ca^2+^-dependent phospholipase A2 (PLA2), which cleaves AA from membrane phospholipids [77,125]. AA is, turn, is converted into EETs, such as 5,6-EET, 8,9-EET, 11,12-EET and 14,15-EET, by cytochrome P450 2C9 or cytochrome P450 2J2 [125]. In particular, 5,6-EET, 8,9-EET and 11,12-EET were shown to stimulate TRPV4-mediated non-selective cation channels and intracellular Ca^2+^ signals in endothelial cells from several vascular beds [191,195,196,197,198]. Luminal application of UTP, which is a selective agonist of P2Y2 receptors, in rat middle cerebral arteries induced TRPV4-mediated Ca^2+^ entry across the luminal and abluminal face of the endothelial monolayer. TRPV4-mediated Ca^2+^ entry, in turn, activated PLA2, whose activation was polarized to the abluminal side, to activate EDH and induce vasodilation [199,200]. Moreover, TRPV4-mediated Ca^2+^ entry was necessary to activate IK_Ca_ and SK_Ca_ channels and induce the vasodilatory response to acetylcholine in mouse posterior cerebral arteries [186]. TRPV4 is, therefore, the most likely candidate to trigger ATP-induced retrograde vasodilation in rat parenchymal arterioles, which is initiated by EETs and involves IK_Ca_, but not SK_Ca_, channels [164]. Intriguingly, EETs mediate vasodilation in rodent cortical arterioles [27,42,201,202]. It has been proposed that NA-induced elevation in astrocyte [Ca^2+^]_i_ engages PLA2 to liberate AA, thereby resulting in EET synthesis, either within astrocytes or adjacent smooth muscle cells [33,42]. Future work is necessary to assess whether: (1) astrocyte-derived EETs also activate endothelial TRPV4 within the NVU; and/or (2) neural activity directly stimulates brain endothelial cells to produce EET synthesis and activate TRPV4. A recent investigation suggested that EDH was responsible for propagating the hemodynamic response to somatosensory stimulation from the capillary bed to upstream penetrating arterioles and pial arteries [7,203]. However, IK_Ca_ and IK_Ca_ currents could not be recorded in mouse brain capillary endothelial cells [204]. These findings strongly suggest that: (1) EDH is restricted to pial arteries and arterioles, but it cannot be activated in the capillary bed; and (2) an alternative mechanism spreads the vasomotor signal from brain capillaries to upstream vessels (see below) [204].

#### 3.5.2. TRPA1, TRPC3 and TRPV3

In addition to TRPV4, TRPA1 channels are also enriched at MEGJs in rat cerebral arteries and may induce EDH in isolated vessels [78]. Accordingly, allyl isothiocyanate (AITC) (mustard oil), a selective TRPA1 agonist, induced local Ca^2+^ entry events, IK_Ca_ channel activation and vasodilation in rat cerebral arteries [205,206]. TRPA1 was physiologically activated by superoxide anions generated by the reactive oxygen species-generating enzyme NADPH oxidase isoform 2 (NOX2), which colocalizes with TRPA1 in cerebral endothelium, but not in other vascular districts [178]. Furthermore, EDH in cerebral arteries may be elicited by Ca^2+^ influx through TRPC3 and TRPV3 channels [78]. TRPC3 is gated by DAG to conduct extracellular Ca^2+^ into vascular endothelial cells upon PLC activation [117,118,138,207]. A recent investigation revealed that TRPC3 contributed to ATP-induced vasodilation in mouse middle cerebral arteries and posterior cerebral arteries by delivering the Ca^2+^ necessary for IK_Ca_ and SK_Ca_ channel activation. IK_Ca_ channels mediated the initial phase of ATP-induced hyperpolarization, whereas SK_Ca_ channels sustained the delayed phase of endothelial hyperpolarization [208]. IK_Ca_ and SK_Ca_ channels in rat brain vessels may also be activated by TRPV3. Accordingly, carvacrol, a monoterpenoid phenol compound highly concentrated in the essential oil of oregano, caused vasodilation by selectively activating TRPV3 in rat isolated posterior cerebral and superior cerebellar arteries. TRPV3-mediated extracellular Ca^2+^ entry, in turn, engaged IK_Ca_ and SK_Ca_ channels to trigger EDH. Unlike TRPV4 and TRPA1, TRPV3 was not specifically localized to MEGJs, but was distributed throughout the endothelial membrane [209]. In addition to carvacrol, TRPV3, as well as TRPA1 are sensitive to several dietary agonists. For instance, TRPV3 may also be activated by eugenol (clove oil) and thymol (found in thyme) [210], whereas allicin (garlic) and cinnamaldehyde induce TRPA1-mediated Ca^2+^ entry [211]. These observations, therefore, suggest that dietary manipulation of TRP channels could be exploited to rescue CBF in cerebrovascular pathologies [6,212]. 

## 4. How to Integrate Endothelial Ca^2+^ Signals in the Current Models of Neurovascular Coupling and Propagated Vasodilation

**Figure 5:**
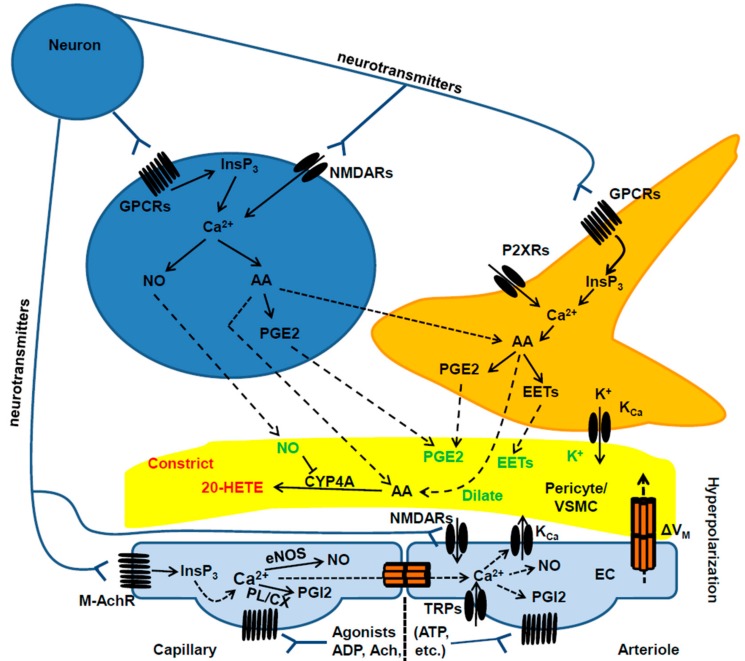
The putative role of endothelial Ca^2+^ signaling in neurovascular coupling. Synaptic activity leads to arteriole and capillary vasodilation by inducing an increase in [Ca^2+^]_i_ in postsynaptic neurons and perisynaptic astrocytes, as shown in Figure 2. Recent evidence indicated that synaptically-released glutamate may activate endothelial NMDARs, thereby eliciting the Ca^2+^-dependent activation of eNOS, in intraparenchymal arterioles. Moreover, acetylcholine may induce Ca^2+^-dependent NO release from brain endothelial cells by initiating the concerted interplay between InsP_3_Rs and SOCE [76]. This local vasodilation may be spread to more remote sites (≈ 500 μm) through the initiation of an interendothelial Ca^2+^ wave, which ignites NO release and PGI2 production as long as it travels along the endothelial monolayer. Moreover, this propagating Ca^2+^ sweep could induce vasodilation by also stimulating K_Ca_ channels and evoking EDH in arterioles. Finally, blood-borne autacoids and dietary agonists could induce vasodilation by, respectively, binding to their specific GPCRs and stimulating multiple TRP channels (TRPC3, TRPV3, TRPV4, TRPA1) to initiate EDH. Abbreviations: 20-HETE: 20-hydroxyeicosatetraenoic acid; AA: arachidonic acid; CX: cyclooxygenases 1 and 2; EETs: epoxyeicosatrienoic acids; GPCRs; G-protein-coupled receptors; InsP_3_: inositol-1,4,5-trisphosphate; K_Ca_: Ca^2+^-activated intermediate and small conductance K^+^ channels; NO: nitric oxide; nNOS: neuronal NO synthase; P450: cytochrome P450; PGE2: prostaglandin E2; PL: phospholipase A2; PLD2: phospholipase D2; TRPs: TRP channels; VSMC: vascular smooth muscle cell.

The role of endothelial Ca^2+^ signaling in NVC has largely been neglected as most authors focused on other cellular components of the NVU, such as neurons, interneurons, astrocytes, smooth muscle cells and, more recently, pericytes [2,4,6,9,30]. However, the literature discussed in the previous paragraphs suggests that the endothelial Ca^2+^ toolkit could play a crucial role in detecting and translating NA into a local vasoactive signal that is then propagated to upstream vessels [7,24]. In order to interpret the precise function of the ion channel network that controls CBF, it is mandatory to understand when and where the hemodynamic response starts. As mentioned earlier, synaptic activity-evoked NVC could be initiated at the capillary level and conducted upstream [12,47,203,213,214,215,216,217]. This hypothesis makes physiological sense as capillaries are closer to active neurons compared to arterioles and could represent the earliest vascular component to detect NA. In addition, endothelial signaling underpins long-range and almost unattenuated propagation (up to 2 mm) of vasomotor responses along peripheral vessels [22,62,63,176,177,218]. Endothelial engagement during NVC could, therefore, fulfill the same function observed in peripheral circulation, i.e., initiating (or contributing to initiate) vasodilation in proximity of the most metabolically active areas and conducting the vasomotor signal to upstream feeding arteries and arterioles to ensure an adequate increase in local blood supply (Figure 5) [62,177].

We believe that there is wide evidence to conclude that acetylcholine, which is liberated by cholinergic afferents emanated from the basal forebrain neurons during alert wakefulness, arousal, learning and attentional effort [28,150], increases CBF by inducing an increase in endothelial [Ca^2+^]_I_, which results in robust NO release [53,76,150,151,153,157]. This model does not rule out the possibility that the sub-population of acetylcholine and NO-synthesizing basal forebrain neurons that send projections onto cortical microvessels could directly control CBF though NO liberation [150,219]; in addition, acetylcholine and NO-synthesizing fibers may also contact NO cortical interneurons, which could contribute to NO-dependent vasodilation [150,220]. Similar to acetylcholine, glutamate could induce NMDARs-mediated NO release from brain endothelium [55,56,127]. Endothelial-derived NO could represent the alternative, i.e., non-neuronal, source of NO supporting vasodilation in cerebellar [141] and cortical [142] parenchymal arterioles. Future work will have to assess whether capillary brain endothelial cells express functional NMDARs and generate NO in response to glutamate stimulation. Intriguingly, the role of glial Ca^2+^ signals [13], which lead to the indispensable release of D-serine in arterioles, and of NO in glutamate-induced capillary dilation have been recently reported in cerebellum [11]. Alternatively, glutamate-induced endothelial-dependent vasodilation could contribute to pre-dilate upstream pial arteries and parenchymal arterioles prior to the retrograde propagation of the vasomotor signal from the capillary bed. Retrograde propagation of the initial vasodilation to upstream cortical vessels still represents a matter of hot debate [4,7,221]. Hillman’s group demonstrated that light-dye disruption of endothelial lining dampens propagation of stimulus (electrical hind paw stimulation)-induced vasodilation in pial arteries in vivo, thereby blunting the increase in CBF [203]. In Section 2.3, we anticipated that endothelial Ca^2+^ signals mediate retrograde propagation of the vasomotor response by engaging two distinct mechanisms: (1) IK_Ca_ and SK_Ca_ channels, which effect the fast component of conducted vasodilation to upstream feeding vessels (i.e., pial arteries and arterioles); and (2) an intercellular Ca^2+^ wave that mediates the slower component of conducted vasodilation by inducing the release of NO and PGI2 from vascular endothelial cells [7,63,64]. EDH cannot, however, be initiated by brain capillary endothelial cells, which lack functional IK_Ca_ and SK_Ca_ channels [204]. Nelson’s group recently revealed that a modest increase in the extracellular K^+^ concentration (≈ 10 mM), which is likely to truly reflect NA, activated brain capillary cell endothelial inward rectifier K^+^ (K_IR_2.1) channels to generate a hyperpolarizing signal that rapidly propagated to upstream arterioles (monitored up to ≈500 μm) to cause vasodilation and increase CBF in vivo [204]. Therefore, it appears that K_IR_2.1, rather than IK_Ca_ and SK_Ca_, channels mediate the fast component of conducted vasodilation in cerebral circulation. Of note, the hyperpolarization conduction velocity was in the same range, ≈2 mm/s, as the propagation speed of retrograde pial artery dilation [204,213]. The secondary slow component of conducted vasodilation is sustained by interendothelial Ca^2+^ waves, as discussed elsewhere [4,7,62]. Earlier work carried out by monitoring endothelial Ca^2+^ with a Ca^2+^-sensitive dye, i.e., Fura-2, showed that local delivery of acetylcholine triggered a Ca^2+^ wave travelling bidirectionally along the endothelium of hamster feed arteries for no less than 1 mm [222]. This intercellular Ca^2+^ wave, in turn, sustained the slow component of conducted vasodilation by promoting NO and PGI2 release [223]. The use of a genetic Ca^2+^ indicator, GCaMP2, confirmed these data in cremaster muscle arterioles in vivo. Accordingly, acetylcholine induced a local increase in endothelial [Ca^2+^]_i_ that activated K_Ca_ channels to induce rapidly-conducting vasodilation at distances >1 mm and travelled along the endothelium as an intercellular Ca^2+^ wave for 300–400 μm. As shown in previous studies [222,223], the intercellular Ca^2+^ wave preceded a secondary vasodilation that was mediated by NO and PGE2 [63]. These findings led to the suggestion that the initial hyperpolarization conducts vasodilation far away (> several millimeters) from the local site of stimulation (including daughter and parent branches), whereas the slower Ca^2+^ wave encompasses only the vessel segments closer to the active region, thereby finely tuning the magnitude and duration of the vasodilatory response [62,63]. A recent study showed that removal of extracellular Ca^2+^ may induce intercellular Ca^2+^ waves in immortalized (RBE4) and primary (from bovine origin) brain microvascular endothelial cells [224]. Although this finding is not sufficient to confirm that interendothelial Ca^2+^ waves may propagate NA-induced local vasodilation to upstream arteries and arterioles, it confirms that brain endothelial cells are able to generate this type of propagating Ca^2+^ signal, which is likely to impinge on InsP_3_Rs [225,226,227]. Future work will have to assess whether: (1) an intercellular Ca^2+^ wave contributes to propagating the hemodynamic response from the capillary bed to feeding vessels; and (2) if so, whether EDH is stimulated by the incoming Ca^2+^ wave in parenchymal arterioles to sustain the vasomotor response.

## 5. Conclusions

Neurovascular coupling is the crucial process to adjust local CBF to the metabolic requirements of active neurons and maintain brain function. Although functional magnetic resonance imaging is routinely employed to monitor the changes in neuronal spiking activity, BOLD signals actually reflect the increases in CBF induced by NA. Therefore, understanding the cellular and molecular bases of NVC is mandatory to interpret the complex relationship between neuronal firing, metabolism and blood flow in both physiological and pathological conditions [6,7,8]. Intracellular Ca^2+^ signaling has long been known to drive NVC by coupling synaptic activity with the production of vasoactive messengers. Emerging evidence, however, suggests that the endothelial Ca^2+^ toolkit could also be recruited by neurotransmitters (i.e., glutamate and acetylcholine) and neuromodulators (e.g., ATP) to induce NO and PGE2 release or activate EDH. Future work will benefit from the availability of transgenic mice selectively expressing a genetic Ca^2+^ indicator, e.g., GCaMP2, in vascular endothelial cells [63,174]. The combination of endothelial GCaMP2-expressing mice with the novel advances in imaging techniques (i.e., two- or three-photon microscopy) will permit assessing whether synaptic activity increases endothelial [Ca^2+^]_i_ in vivo. It will also be important to decipher the role played by IK_Ca_ and SK_Ca_ channels in the hemodynamic response to NA. Earlier work showed that EDH is involved in local and retrograde vasodilation in parenchymal arterioles and middle cerebral arteries, but is absent in capillaries, where functional hyperemia is likely to initiate in response to sensory stimulation. The local vasodilation induced by NA increases the strength of laminar shear stress acting on vascular endothelium, a mechanism that leads to TRPV4-dependent NO release and EDH in peripheral circulation [191,228]. There is, however, conflicting evidence regarding flow-induced vasodilation in pial arteries and parenchymal arteries in the brain [229,230,231]. Nevertheless, EDH could be exploited by dietary manipulation to treat the vascular dysfunctions associated with aging and neurodegenerative disorders, which cause cognitive impairment by halting CBF [2,3,6,212]. For instance, NVC is severely impaired in Alzheimer’s disease due to a defect in NO release [217,232,233]. A recent study revealed that the intracellular Ca^2+^ toolkit is severely compromised in rat brain microvascular endothelial cells exposed to amyloid-beta (Aβ) peptide [93], whose accumulation in brain parenchyma and in the cerebrovasculature represents a major pathogenic factor in AD [4]. Future work will have to assess whether dietary and/or pharmacological manipulation is able to rescue or halt functional hyperemia in these subjects by activating the TRP channels (i.e., TRPC3, TRPV3, TRPV4 and TRPA1), which are coupled to EDH in the brain. An emerging area of research for NVC is represented by the vasodilating role of mitochondrial Ca^2+^ in brain microvascular endothelial cells. A recent series of studies revealed that mitochondrial depolarization, induced by BMS-291095 (BMS), an opener of mitochondrial ATP-dependent K^+^ (K_ATP_) channels, prevented Ca^2+^ accumulation within the mitochondrial matrix, thereby resulting in a large increase in [Ca^2+^]_i_ in rat cerebral arteries endothelial cells. This mitochondrial-derived Ca^2+^ signal, in turn, induced eNOS activation, NO release and endothelium-dependent vasodilation [95]. Intriguingly, subsequent work demonstrated that mitochondrial-dependent NO release and vasodilation were impaired in cerebral arteries of obese Zucker rats [234]. The physiological stimulus responsible for mitochondrial-induced NO release in cerebral endothelium is yet to be elucidated, but it could be implicated in many other cerebrovascular disorders. Finally, astrocyte-released AA is the precursor of multiple vasoactive messengers, such as PGE2, EETs and 20-HETE, which are all involved in NVC, although their contributions depend on the vascular segment and/or the brain area. However, AA was shown to induce Ca^2+^-dependent NO release by directly activating TRPV4 in several types of endothelial cells [54,235]. Some evidence suggested that AA was able to induce Ca^2+^ signals in human brain microvascular endothelial cells [94]. Given the key role of this lipid mediator within the NVU, we predict that future work will unveil that AA-dependent endothelial Ca^2+^ signals may also contribute to NVC.

## Abbreviations

20-HETE	20-hydroxyeicosatetraenoic acid
AA	arachidonic acid
AMPA	a-amino-3-hydroxy-5-methyl-4-isoxazol epropionic acid
BK_Ca_	large-conductance Ca^2+^-activated K^+^ channels
BOLD	blood oxygenation level dependent
CaM	Calmodulin
CBF	cerebral blood flow
cGMP	cyclic guanosine-3′,5′-monophosphate
CICR	Ca^2+^-induced Ca^2+^ release
CYP4A	cytochrome P450 4A
DAG	Diacylglycerol
EDH	endothelial-dependent hyperpolarization
EETs	epoxyeicosatrienoic acids
EL	Endolysosomal
eNOS	neuronal NO synthase
ER	endoplasmic reticulum
GPCRs	G-protein-coupled receptors
IK_Ca_	intermediate-conductance Ca^2+^-activated K^+^ channels
InsP_3_	inositol-1,4,5-trisphosphate
InsP_3_Rs	InsP_3_ receptors
K_Ca_	Ca^2+^-activated K^+^ channels
K_ir_	inward rectifying K^+^ channels
M-AchRs	muscarinic acetylcholine receptors
MCU	mitochondrial Ca^2+^ uniporter
MEGJs	myo-endothelial gap-junctions
mGluRs	metabotropic glutamate receptors
NA	neuronal activity
NAADP	nicotinic acid adenine dinucleotide phosphate
NMDA	*N*-methyl-d-aspartate
NMDARs	NMDA receptors
NO	nitric oxide
nNOS	neuronal NO synthase
NOX2	NADPH oxidase isoform 2
NVC	neurovascular coupling
NVU	neurovascular unit
O_2_	Oxygen
P450	cytochrome P450
PGE2	prostaglandin E2
PIP_2_	phosphatidylinositol-4,5-bisphosphate
PLA2	phospholipase A2
PLD2	phospholipase D2
RyRs	ryanodine receptors
SERCA	sarco-endoplasmic reticulum Ca^2+^-ATPase
SK_Ca_	small-conductance Ca^2+^-activated K^+^ channels
SOCE	store-operated Ca^2+^ entry
Stim	stromal interaction molecule
TPCs	two-pore channels
TRPs	TRP channels
TRPA1	ankyrin TRP 1
TRPC	canonical TRP
TRPV4	vanilloid TRP 4
VDACs	voltage-dependent anion channels
VSMC	vascular smooth muscle cells
